# Cancer incidence in English children, adolescents and young people: past trends and projections to 2030

**DOI:** 10.1038/bjc.2017.341

**Published:** 2017-11-02

**Authors:** Francesca Pesola, Jacques Ferlay, Peter Sasieni

**Affiliations:** 1 Wolfson Institute of Preventive Medicine, Queen Mary University of London, Charterhouse Square, London EC1M 6BQ, UK; 2 Section of Cancer Surveillance, International Agency for Research on Cancer, Lyon 69372, France

**Keywords:** childhood, young adults, adolescence, cancer, incidence, projections, APC

## Abstract

**Background:**

Estimating the future incidence of cancer is important to establish sufficient service provision, however, work in this area is limited for cancer in children, adolescents, and young adults (aged 0–24).

**Methods:**

Age-period-cohort models were applied to cancer incidence rates for the period 1971–2013 in England. This allowed us to extrapolate past trends to 2030. We used the appropriate cancer classification developed for cancers in children and young adults, which are analysed as two separate groups to capture inherent differences.

**Results:**

The data set consisted of 119 485 records (55% among 15+ years group). Overall, cancer rates have increased over time and are expected to continue to rise into the future. Of particular interest is the increase in rates of germ cell tumours (in males) and carcinomas (in females) in young adults, since their rates are projected to further increase over time.

**Conclusions:**

The estimated future incidence rates provide a baseline for different cancer subtypes, which will allow policymakers to develop a contingency plan to deal with future demands.

Cancers in children and young adults (aged 0–24) are rare as they account for approximately 1.5% of all cancers (Cancer Research UK, 2015a); however, cancer represents a leading cause of death in this age group (Cancer Research UK, 2015b). Estimating the future incidence of cancer is important to set up services, effectively allocate resources, establish sufficient service provision, and define a baseline against which we can assess efficacy of future interventions (National Institute for Health and Clinical Excellence, 2005).

A recent study estimated the incidence of adult cancers in the UK up to 2030 (Mistry *et al*, 2011). These projections indicated that the number of cancers would increase by 55% and 35% in men and women, respectively, reflecting the growing and ageing UK population (Mistry *et al*, 2011). Owing to the different nature of cancers observed in children, teenagers, and young adults compared to adults, it is necessary to undertake projection analyses of cancer incidence specifically in this age group.

A study looking at the trends of cancer incidence in childhood and adolescence (aged 0–19 years) across 19 European countries found that there had been an increase in cancer incidence between 1970 and 1999 (Steliarova-Foucher *et al*, 2004). Similarly, a more recent study, using data from 62 countries, showed an increase in cancer incidence rates from the 1980s and the period 2001–2010 (Steliarova-Foucher *et al*, 2017). Using data published annually by the Office for National Statistics (ONS, 2014), Cancer Research UK offers a graphical representation of the increased cancer incidence from the 1960s in children (Cancer Research UK, 2010) and adolescents (Cancer Research UK, 2013). Nonetheless, no study to date has systematically explored past trends or estimated cancer incidence among children and young adults over the next decades in England. We aimed to fill this gap in the literature using Cancer Registry data recorded in England from 1971 and following the approach implemented by Mistry *et al* (2011). A major strength of the data set is the long time frame (1971–2013) and having data that allows for finer inspection by age, year of birth, and type of cancer. The findings will allow us to obtain an estimate of the future burden of cancer among children and young people and, hence, this work should allow health providers to develop a contingency plan.

**Research aims.** Using data on the incidence of cancer in England from 1971 to 2013, we aim to (a) explore past trends; (b) make cancer incidence projections up to 2030 for children and young adults.

## Materials and methods

### Data source

Cancer data, previously recorded by nine population-based regional Cancer Registries in England, were provided by Public Health England. The data contained 214 851 malignant as well as benign and *in situ* cancers diagnosed in England between 1971 and 2013. Different coding systems were used in this time window. The information consisted of cancer topology, morphology, and behaviour. Data were broken down by sex, year of diagnosis, and single year of age at diagnosis. The National population estimates (1971–2014) and 2014-based population projections to 2030 for England broken down by sex and single year of age were obtained from the Office for National Statistics (ONS, 2014).

From 1971 to 1978, topography was recorded using the International Classification of Diseases 8th Edition (ICD-8) while morphology was coded using the Manual Of Tumour Nomenclature And Coding (MOTNAC). Between 1979 and 1989, topography was recorded using ICD-9 and morphology using 4-digit ICD-O-1 codes; the latter did not include the fifth digit that codes behaviour. Thus, we derived the behaviour information based on the cancer topography. Specifically, behaviour ‘3’ (malignant) was assigned to records with topography codes 140.0 to 208.9, a behaviour ‘0’ (benign) was allocated to topography codes 210.0 to 229.9, a behaviour ‘2’ (*in situ*) to topography 233.0 to 234.9 and finally a behaviour ‘1’ (borderline malignancy) to topography codes 235.0 to 239.9. Between 1971 and 1989, 3457 records were coded with a four-digit code with values ranging between 6000 and 7999. Based on published information, ‘2000’ was added to these codes to obtain MOTNAC codes (Dickinson *et al*, 2001). Between 1990 and 1994, topography was recorded using ICD-9 and morphology using five-digit ICD-O-1 codes. Finally, between 1995 and 2013 topography was recorded using ICD-10 and morphology using ICD-O-2.

### Case definition

Cases were restricted to individuals aged 0–24 years, who were classified as children (aged 0–14 years), and adolescents and young adults (15–24 years) and were diagnosed with a primary cancer in England between 1971 and 2013. Cases were categorised into main cancer groups using topography, morphology, and behaviour information.

In order to classify the records into main subtypes, topography and morphology codes were converted to ICD-O-3, which is used to classify cancers in the International Classification of Childhood Cancer (ICCC3) (Steliarova-Foucher *et al*, 2005) and Adolescence and Young Adulthood (AYA) (Barr *et al*, 2006) classification systems. The latter was originally developed using ICD-O-2 codes but SEER offers coding instructions which use ICD-O-3 codes (SEER, 2008). These two coding schemes reflect the fact that these two age groups are affected by different kind of neoplasms (Barr *et al*, 2006).

We are interested in malignant tumours (behaviour=3); however, when coding cancers of the central nervous system (CNS) and miscellaneous intracranial and intraspinal neoplasms as well as intracranial and intraspinal germ cells, all behaviours except *in situ* (behaviour=2) are included. In total, 119 485 (65 412, 55% among the 15+ age group) records were successfully recoded using ICCC3 and AYA.

### Data analysis

The main outcomes from our analyses are trends and projections of incidence rates for different cancer types. An age-period-cohort model approach assuming a Poisson distribution of cancer events was implemented to analyse the data to model past trends and estimate future incidence rates up to 2030, using a modified version of the apcspline command in Stata 13 (Sasieni, 2012). The analyses were conducted separately for males and females to identify potential sex differences. The basic APC model is:


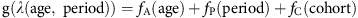


where *λ* is the incidence rate as a function of age and calendar period, g is the ‘link’ function and *f*_A_, *f*_p_ and *f*_C_ are functions of age, period (i.e., year of incidence) and cohort (i.e., year of birth). In the present analysis, we use 1-year period and single year of age, for period and age respectively. In our model, we fit the data using the log link; however, we compared the fit of this model to a model using the one-fifth power function (g(*x*)=*x*^1/5^), owing to the fact that the latter offered a good fit to the data when looking at the adult population (Mistry *et al*, 2011). Estimates will be presented based on the results obtained using the log link, unless the model using the power link offers a better fit, as indicated by the Akaike Information Criterion (AIC) model fit (Bozdogan, 1987). For the functions, *f*_A_, *f*_p_ and *f*_C_, we use natural cubic splines as they offer greater flexibility and more realistic projections than using a step function, as changes can be expected to occur smoothly rather than in sudden jumps.

Finally, we applied a damping factor to the drift (i.e., the linear trend over time) when extrapolating to the future. This takes into account the fact that current trends are not expected to continue over time and their effect will wane (Moller *et al*, 2002). We set the damping factor equal to 0.92 so that we reduce the drift by 8% for each year following the last observation. This damping factor was chosen so that the drift will be approximately half of that during the observation period after 8 years (Sasieni, 2012). This is similar to the linear damping used by NordPred and previously validated for adult populations (Moller *et al*, 2003). This is also the default setting by the apcspline command developed by Sasieni (2012). We calculated prediction intervals to capture the variance of the parameters in the model and variation in the estimated future cases using the approach described by Hakulinen and Dyba (1994).

We compared a number of models (i.e., null, age-drift, age-period, age-cohort and age-period-cohort) to identify the one that best fit our data using the Pearson’s chi-squared statistics as a measure of goodness of fit. We did not use significant testing. Indeed, the Pearson chi-squared statistic provides a measure of variation such that models with smaller values offer a better fit to the data. Cancer incidence rates are presented per million person years. These incidence rates were converted to actual number of cases by multiplying them by the population projections (in millions) for England by sex, 1-year age group and year. Sensitivity analyses were conducted to estimate the number of cancers using population projections under low and high migration scenarios to compare them to the number from the primary analysis (ONS, 2014). Finally, incidence rates were standardised using the European standard population (EUROSTAT, 2013) to calculate age-standardised rates (ASRs). ASR for years 1989, 2009, and 2029 are based on a 3-year average; for example, ASRs for 1989 are based on averaged incidence rates across years 1988, 1989, and 1990. The rates for 1989 and 2009 are for the observed data while those of 2029 are based on the predictions for the selected model using the log link. Further sensitivity analyses were conducted to explore the impact of the model assumptions on the projected rates (i.e., magnitude of damping factor and inclusion of a shorter time frame).

## Results

**Figure 1:**
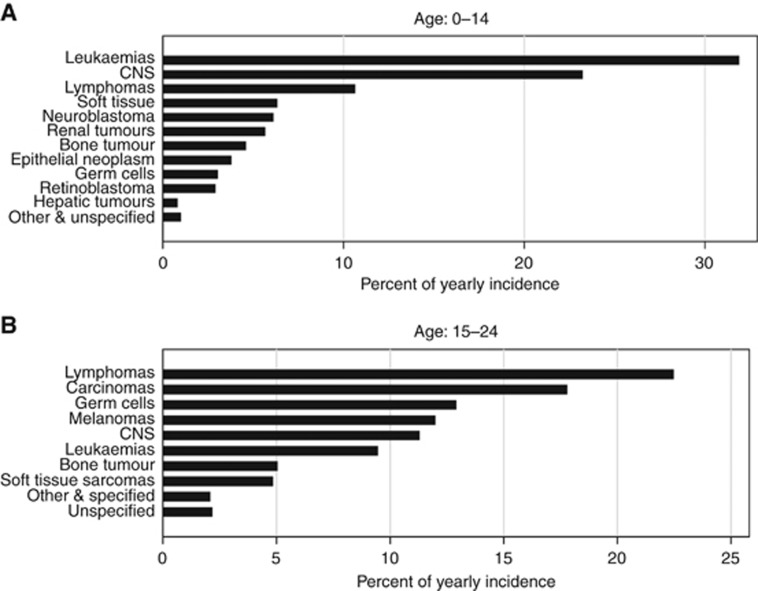
**Cancer subtypes.** Cancer distributions among (**A**) children and (**B**) adolescents and young adults diagnosed between 1971 and 2013.

Figure 1 shows the breakdown of the different cancer types observed in children and in teenagers and young adults. The data show different cancer subtypes are observed in the two age groups and, hence, confirms the importance of looking at them separately.

### Fine tuning the APC model

Based on AIC, the model using the power-link model did not fit the data better than the model using the log link (Supplementary Table 1). Therefore, projections presented in this section are based on the model using the log link. Moreover, the model fit results showed that an age drift (A-drift) model offered as good a fit as more complex models to the data for all cancers among children (Supplementary Table 1). Hence, we selected the A-drift model as it is more parsimonious than those including a non-linear period and/or cohort effect. Similarly, for cancers in the older age group, we found that the A-drift model offered a reasonable fit to the data for all cancers but Hodgkin’s lymphomas and carcinomas, which were better captured by an AC model and an AP model for germ cells. Hence, the more complex model was used to analyse data for these three cancers.

Figures 3, 4, 5 show the trends (1971–2013) and projections (2014–2030) of cancer incidence rates per million by sex and 5-year age group for the main cancer categories. The lines are the projections based on the log-link (i.e., the best fitting model). The dots are the rates as observed between 1971 and 2013. The black lines are the estimated cancer numbers. In the graphs, years are grouped in 3-year periods (e.g., 1971–1974) to enhance smoothing.

### Age effect

**Figure 2:**
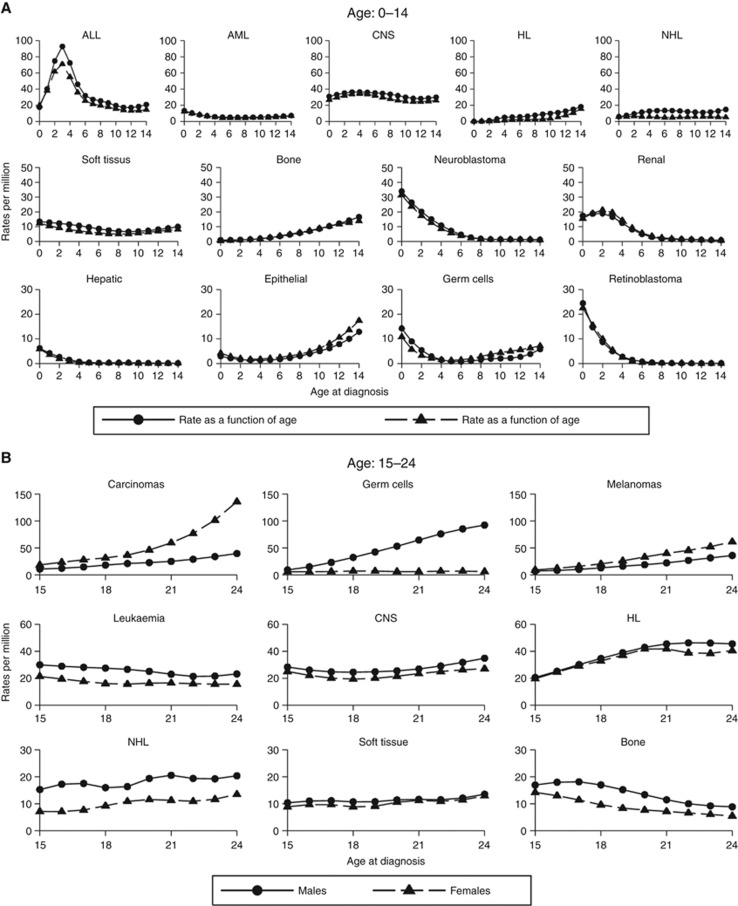
**Age effect.** Rate per million for (**A**) children and (**B**) teenagers and young adults.

The left panel of Figure 2 shows how cancer incidence in children varies as a function of age for the different subtypes. Leukaemia is the most common cancer among children (33%). The most common sub-type is the acute lymphoblastic leukaemia (ALL) which accounts for 77% of all cancers and peaks between ages 3 and 5. Acute non-ALL cancers are mostly acute myeloid leukaemia (AML; 15%). Hence, it is worth exploring future projections for ALL and AML, separately. Tumours of the CNS are the second most common cancer (23%) and incidence peaks before the age of 5. Similarly, the incidence rates of embryonal tumours (i.e., neuroblastomas), retinoblastomas, and renal cancers appear to peak at younger ages (<5). Lymphomas are the third most common cancers in this age group and account for 11% of all cancers in childhood. Non-Hodgkin’s lymphoma is the most common subtype (59%) and rates appear stable across the age range. The incidence rate of Hodgkin’s lymphomas (HL; 41%) increases over age and peaks at age 12–14.

Figure 2 (right panel) shows how cancer incidence in adolescents and young adults vary as a function of age for the different subtypes. In this older age group, leukaemia accounts for just under 10% of all cancers and is not as common as among children. Instead, lymphomas are the most common cancer (23%) across both sexes. HL constitutes 72% of all observed lymphomas and its incidence rate peaks in the early twenties and appears stable in this period. Carcinomas and germ cells tumours are the second and third most common cancers in this age group but sex differences exist. Indeed, carcinomas are 2.4 times more common in females than males and incidence rates increase with age with peaks around ages 23–24. Similarly, among males rates increase with age and peak at ages 23–24. For germ cells, 83% of cases are observed in males and 85% of all records were cancers of the gonadal germ cells and trophoblastic neoplasm. Incidence rates appear to increase linearly from age 15 to 24. Similarly, for melanomas the incidence rates appear to increase with age while for bone tumours and leukaemia the rates decrease with age as incidence peaks around age 15–17 and, subsequently, decrease.

### Future projections

**Figure 3:**
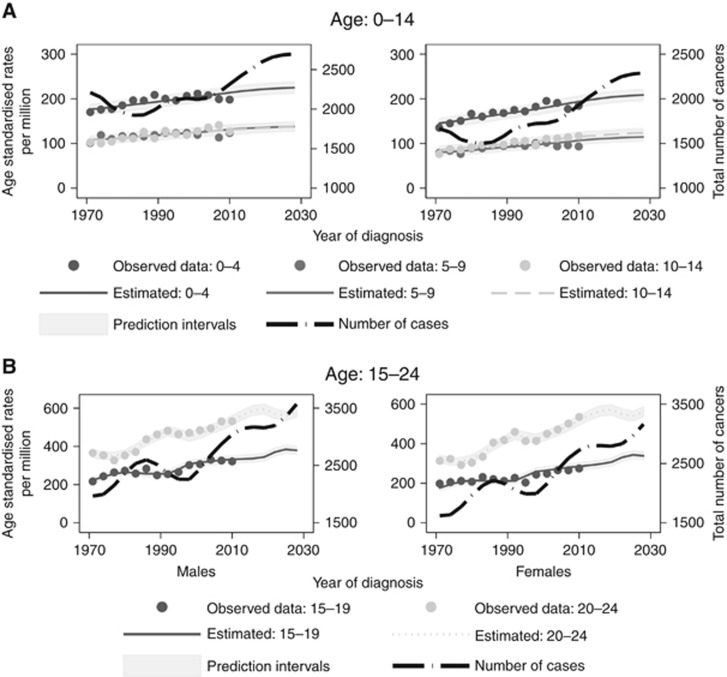
**Rates per million (European standard) for cancer incidence broken down by sex for children (A) and adolescents and young adults (B).** Number of cancers (for all ages) are also presented by the black long-dash line.

Figure 3 shows the trends (1971–2013) and projections (2014–2030) by sex and 5-year age group for all sites combined for children and young adults, separately. The dots indicate the observed rates while the lines represent the estimated rates by age group. The figures suggest that cancer rates have increased in the observed period and are anticipated to further increase up to 2030.

**Figure 4:**
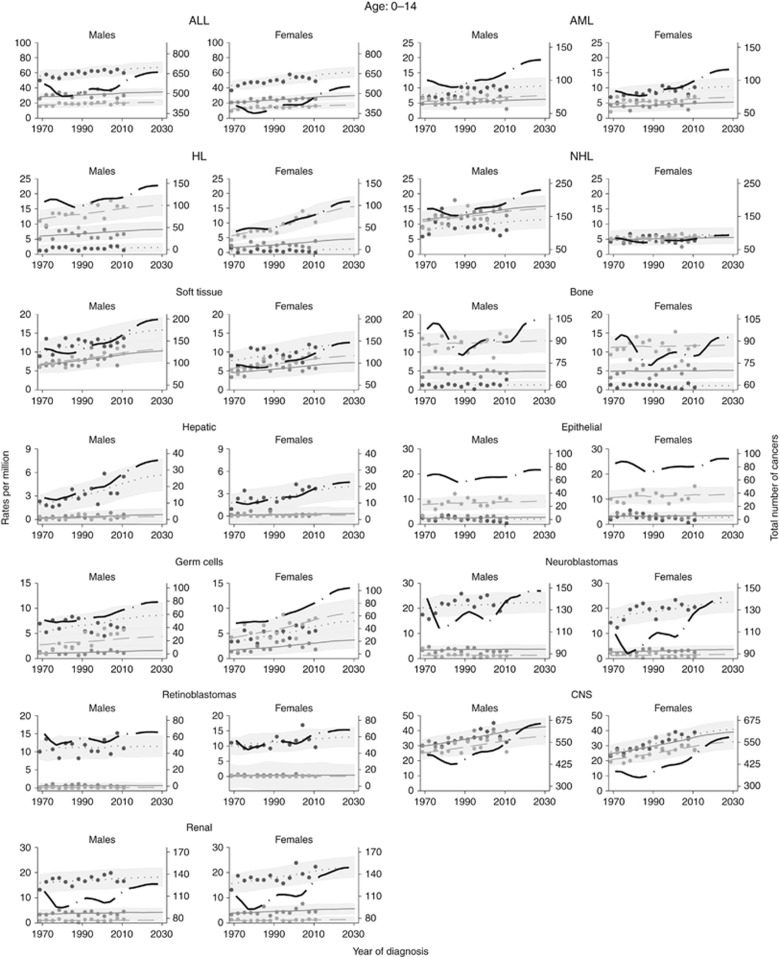
**Trends and projections by cancer subtype broken down by sex among children (continuous line ages 0–4, dotted line 5–9, and dashed line ages 10–14).** Number of cancers (across all ages) are also presented by the black long-dash line.

**Figure 5:**
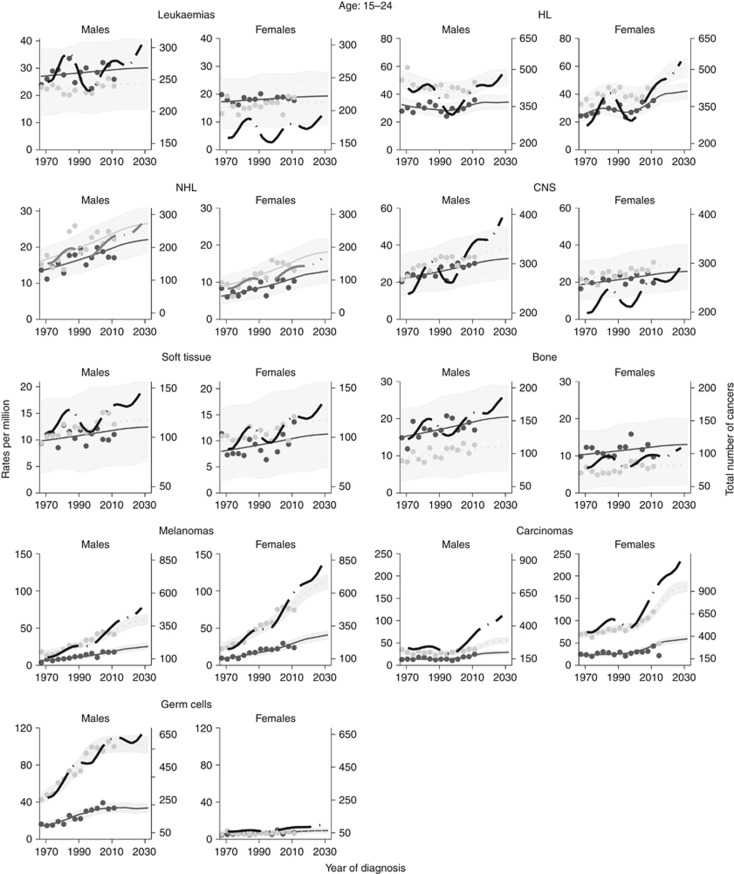
**Trends and projections by cancer subtype broken down by sex among teenagers and young adults (continuous line ages 15–19 and dotted line ages 20–24).** Number of cancers (across all ages) are also presented by the black long-dash line.

Figures 4 and 5 show the trends and projections for the different cancer sub-types among children and young adults, respectively. The rates are broken down by age group and presented separately for males and females.

Table 1 shows the age-standardised incidence rates (ASR) and numbers of cancers for all sub-types and all cancers combined for children and young adults, respectively. The results presented in Table 1 indicate that rates for all cancer sites combined have increased since 1989 across both sexes in children. Additionally, the ASRs are expected to further increase by 8% and 17% in males and females, respectively from 2009 to 2029. According to ASRs, by 2029 cancer incidence rates are expected to increase by at least 1% for all cancers but retinoblastoma and bone for males and renal and bone for females, where we are predicting a decrease.

Similarly, rates for all cancer sites combined have increased since 1989 across sex among adolescents and young adults (Table 1). Moreover, the ASRs for all cancers are expected to further increase by 9% and 13% in males and females, respectively, by 2029. Rates are projected to increase by 1% or more for all sites but leukaemias for males and females. The projected numbers of cancers under the standard population projections do not differ greatly from the number of cancers estimated using population projections under the low and high migration scenarios (Supplementary Figure 1).

### Sensitivity analysis

A series of sensitivity analyses was conducted to explore the potential effect on the model projections as some of the assumptions are modified. This was done to account for the fact that some of the assumptions are subjective and model results vary as we modify the criteria applied to the model.

To account for the fact we are not expecting past trends to continue into the future, we applied a damping factor to the drift factor which halves the drift after 8 years. This damping factor was selected as it had been previously found to offer a good fit to cancer incidence data in adults (Moller *et al*, 2003; Mistry *et al*, 2011); however, we were interested in assessing how projections to 2030 would vary depending on the damping factor applied to the projections. Specifically, we used the age-drift model to project cancer incidence to 2030 applying either no damping or a damping factor which doubled the current damping so that after 8 years the drift was approximately three-fourth of the one observed in the last year of available data. These results are presented in Supplementary Figures 2 and 3. As expected, using the ‘double’ damping factor produced greater flattening. In contrast, not applying a damping factor simply implies past trends will carry on into the future, although we are not expecting this to be the case. Overall no major discrepancies are observed in the projections.

In the main analysis, we included all data available from 1971 to 2013. We also conducted a series of sensitivity analysis to ascertain how projected rates would vary if only more recent data (i.e., trends) were to be included. To achieve this, we included data from 1981 or 1991 in the model and compared it with the model which included the entire time period. The results, presented in Supplementary Figures 4 and 5, show that projected rates did not differ greatly as a function of the data used in the model.

To ascertain the extent to which changes in projected numbers are due to actual increase in risk rather than population, we took the estimated number of cancers obtained using our main model and compared them against numbers obtained by combining current rates (2011–2013) and population projection in 2030 (i.e., approach 1) as well as against numbers estimated by combining projected rates in 2030 and current population estimated in 2013 (i.e., approach 2; Supplementary Table 2). The results show that the number of estimated cancers using these alternative approaches did not greatly differ from those obtained in the main analysis. We, however, noticed a higher number of cancers were estimated among adolescents and young adults using approach 2 (i.e., projected rates applied to current population).

## Discussion

We used an age-period-cohort model to explore past trends and estimate future rates of cancer incidence in England. Our results indicate that incidence rates have increased in the observed period and they will continue to increase between 2014 and 2030, across both sexes and age groups. These results are in line with those observed in the ACCIS data set, which also showed cancer rates have increased between the 1970s and 1990s among young people aged 0–19 in Europe (Steliarova-Foucher *et al*, 2004). Our study extends previous findings by using past trends to estimate future rates to 2030 for different cancer subtypes and age groups. The most common cancers in children were ALL, CNS, and lymphomas. Overall, incidence of all cancers, apart from retinoblastoma and cancer of the bones among males, have shown a steady increase in incidence rates over the observed years. This increase is expected to further continue to the year 2030, assuming the underlying risk factors continue and the model assumptions are true.

The most common cancers in young adults were lymphomas, carcinomas, and germ cells, whose rates have increased in the observed period as for all other cancers. Carcinomas are most common in females and the increase in rates is mostly driven by an increase in the incidence of carcinomas of the cervix and thyroid. This pattern had previously been observed in cancer registry data from 1979 to 2013 in England when focusing on individuals aged 13 to 24 (Alston *et al*, 2008). These researchers suggested the increase in cervical cancer incidence rate is due to higher incidence of HPV infection in this age group. Based on current evidence (Cuzick *et al*, 2010; Joura *et al*, 2015), a reduction of this rate is expected following the introduction of the national HPV vaccination (of adolescent girls) in 2008 (Cuzick *et al*, 2010; Joura *et al*, 2015).

Research, using cancer registry data for the North of England, similarly found an increase in carcinomas of the thyroid (Cotterill *et al*, 2001). The authors concluded this increase may be partly due to improved diagnostics but also by increased exposure to radiations. In particular, the authors consider the impact of the Chernobyl accident as they only consider data between 1987 and 1997. Nonetheless, the increase in cancer rates observed in more recent years is more likely to be due to improved diagnostics rather than exposure to radiations (Vaisman *et al*, 2015).

In young adults, we also found an overall increase in germ cell tumours, which are mostly observed in males as also observed in the ACCIS data set (Steliarova-Foucher *et al*, 2017). Our projections are reassuring as they indicate that rates’ changes will slow down in the future and plateau for this cancer type by 2029. We also found an increase in the incidence rate of melanomas in the older age group across both sexes. These results are in line with data which indicate increased rates in the overall population as well as younger age groups (age 10–29) in England (Wallingford *et al*, 2013).

Our model appears to fit the data well. The major advantage of our analysis is the fact we have annual data broken down by single year of age and sex over a 40-year window which permit predictions using modelling; however, there are some limitations. The main limitation of our study is the fact that some cancers are fairly rare (e.g., retinoblastoma in children) and past trends noisy, which renders our task harder and requires cautious interpretation of our results. Nonetheless, the past trends estimated in the current paper can be used as a starting point for more detailed epidemiological studies. Another limitation common to all future projection analyses is that they are dependent on the assumptions made. Hence, we conducted a series of sensitivity analyses to assess how varying the assumptions applied to the model (e.g., damping factor) influenced the projected cancer incidence. We applied a damping factor to the future projections as we are not expecting current trends to continue into the future. The selection of the damping factor is based on evidence from analysis of cancer data in adults that showed applying a damping factor, which halved the drift within 8 years of the last year with observed data, offered a good fit to cancer data in adults (Moller *et al*, 2003; Mistry *et al*, 2011). Our sensitivity analysis showed varying the damping factor did not greatly modify our results and, overall, showed that a factor of 0.92 offered a good fit to the data for children, adolescents and young adults.

As the basic assumption is that past trends will continue into the future, we were interested in assessing whether projections would vary depending on whether data from the whole time period were included as opposed to more recent years and, therefore, trends. These sensitivity analyses showed that projections did not vary greatly depending on the time period included in the model. Overall, our sensitivity analysis suggests the assumptions of our main model are sensible and the projected rates reliable.

The increasing rates observed in data, assumed to continue into the future, may result from improved recording of cancer incidence as well as improved diagnostics; however, since the increase is not observed uniformly across age groups (or sex), we are confident some of the change over time is real and due to environmental and behavioural changes. Additionally, sensitivity analysis exploring the impact of changes in population suggested increased numbers of cancers are due to higher rates in the figure rather than being purely driven by an increase in the population.

Using cancer registry data up to 2013, we observed a general increase in incidence rates of cancers among children and young adults, although greater in the older age group. The projections of cancer incidence presented in this paper will provide an up-to-date baseline for future planning of cancer resources for all cancer subtypes.

## Figures and Tables

**Table 1:** ASR per million (European population) and number of cancers among children (0–14 years) and adolescents and young adults (15–24 years)

	**Males**										**Females**									
	**ASR**					**Number of cases**					**ASR**					**Number of cases**				
				**% change 2009–2029**					**% change 2009–2029**					**% change 2009–2029**					**% change 2009–2029**	
	1989	2009	2029	Tot	Ann	1989	2009	2029	Tot	Ann	1989	2009	2029	Tot	Ann	1989	2009	2029	Tot	Ann
**Children (age: 0–14)**																				
																				
Leukaemias	7.6	7.5	8.1	8%	0.3%	226	231	278	20.0%	0.7%	6.1	6.4	7.2	13%	0.4%	175	188	236	25%	0.8%
HL	0.1	0.1	0.1	10%	0.3%	26	38	48	26.1%	0.9%	0.1	0.1	0.1	67%	1.9%	15	19	36	92%	2.4%
NHL	0.2	0.2	0.2	12%	0.4%	58	60	77	28.6%	0.9%	0.1	0.1	0.1	19%	0.7%	26	23	31	35%	1.1%
CNS	5.3	5.7	6.5	13%	0.4%	152	171	219	28.0%	0.9%	4.4	4.9	6.0	21%	0.7%	121	141	193	36%	1.2%
Neuroblastomas	1.5	1.2	1.4	17%	0.6%	48	39	49	26.9%	0.9%	1.4	1.1	1.4	27%	0.9%	42	35	47	36%	1.1%
Retinoblastomas	0.5	0.8	0.6	−22%	−0.9%	15	26	22	−17.0%	−0.7%	0.5	0.6	0.7	22%	0.7%	14	18	23	29%	0.9%
Renal	1.1	1.1	1.2	14%	0.5%	34	34	42	25.4%	0.8%	1.2	1.2	1.5	21%	0.7%	35	38	50	31%	1.0%
Hepatic	0.2	0.2	0.3	51%	1.5%	5	7	12	63.6%	1.8%	0.1	0.1	0.2	53%	1.6%	4	5	8	62%	1.8%
Bone	1.3	1.2	1.0	−15%	−0.6%	34	35	35	−1.8%	−0.1%	1.0	1.1	1.0	−12%	−0.5%	26	30	31	2%	0.1%
Soft tissue	1.5	1.7	1.9	11%	0.4%	43	53	66	25.1%	0.8%	1.1	1.4	1.5	10%	0.4%	32	40	49	22%	0.7%
Germ cells	0.5	0.7	0.8	12%	0.4%	16	21	26	24.8%	0.8%	0.7	0.8	1.1	28%	0.9%	19	24	35	43%	1.3%
																				
All cancers	23.5	24.5	26.3	8%	0.3%	690	744	899	20.8%	0.7%	19.4	20.1	23.5	17%	0.6%	546	584	763	31%	1.0%
**Adolescents and young adults (age: 15–24)**																				
																				
Leukaemias	3.0	3.2	3.1	−2%	−0.1%	92.3	95.3	101.5	6%	0.3%	1.8	2.1	2.1	−2%	−0.1%	52.3	62.0	64.0	3%	0.2%
HL	4.7	4.7	4.9	5%	0.2%	145.0	141.3	160.0	13%	0.6%	4.4	4.5	5.8	29%	1.3%	134.0	131.7	177.5	35%	1.5%
NHL	2.5	2.4	2.8	14%	0.7%	78.3	73.3	90.5	23%	1.1%	1.2	1.1	1.8	57%	2.3%	35.7	33.3	54.8	64%	2.5%
CNS	3.4	3.6	4.0	11%	0.5%	105.0	109.7	131.1	20%	0.9%	2.8	3.0	3.2	5%	0.2%	84.0	89.3	97.9	10%	0.5%
Bone	1.5	1.7	1.9	10%	0.5%	45.3	51.7	61.7	19%	0.9%	0.9	0.9	1.2	24%	1.1%	25.7	27.7	36.3	31%	1.4%
Soft tissue	1.4	1.4	1.5	4%	0.2%	44.0	43.3	49.0	13%	0.6%	1.4	1.3	1.4	13%	0.6%	41.3	37.7	44.5	18%	0.8%
Germ cells	5.9	7.7	7.8	1%	0.1%	184.7	230.3	252.0	9%	0.4%	0.7	1.0	1.1	3%	0.2%	20.0	30.3	32.8	8%	0.4%
Melanomas	2.3	4.0	5.0	25%	1.1%	73.3	120.0	162.5	35%	1.5%	4.3	6.2	8.9	43%	1.8%	131.7	181.0	269.3	49%	2.0%
Carcinomas	2.2	3.2	4.9	52%	2.1%	68.0	97.0	159.1	64%	2.5%	6.1	8.5	13.7	61%	2.4%	187.3	249.7	415.6	66%	2.6%
																				
All cancers	28.7	33.6	36.6	9%	0.4%	892.0	1,010.0	1,189.4	18%	0.8%	25.1	30.3	34.4	13%	0.6%	757.3	891.3	1,054.3	18%	0.8%

Abbreviations: Ann=Annual; ASR=age-standardised rate; CNS=central nervous system; HL=Hodgkin’s lymphoma; NHL=non-Hodgkin’s lymphoma; Tot=total.
