# Supplementary material for: Cancer incidence in English children, adolescents and young people: past trends and projections to 2030
Source: Br J Cancer. 2017 Nov 2;117(12):1865–73. doi: 10.1038/bjc.2017.341 (PMC5729467; doi:10.1038/bjc.2017.341)
Supplement: Supplementary Table 1 [file bjc2017341x1.docx]

**SUPPLEMENTARY TABLE 1**

1. **Model fit indices: Children (age 0-14 years)**

|  | | | **Males** | | | | **Females** | | | |
| --- | --- | --- | --- | --- | --- | --- | --- | --- | --- | --- |
|  |  |  | **LOG** | | **POWER** | | **LOG** | | **POWER** | |
|  | **Model** | **df** | **Chi^2^** | **AIC** | **Chi^2^** | **AIC** | **Chi^2^** | **AIC** | **Chi^2^** | **AIC** |
| ALL | Null | 644 | 6.26 | 9.63 | 6.26 | 9.63 | 4.74 | 8.05 | 4.74 | 8.05 |
| ALL | AD | 638 | 1.18 | 5.31 | 1.17 | 5.30 | 0.92 | 4.78 | 0.92 | 4.78 |
| ALL | AP | 636 | 1.17 | 5.30 | 1.16 | 5.29 | 0.90 | 4.77 | 0.91 | 4.77 |
| ALL | AC | 636 | 1.17 | 5.30 | 1.16 | 5.30 | 0.91 | 4.78 | 0.92 | 4.78 |
| ALL | APC | 634 | 1.17 | 5.30 | 1.16 | 5.30 | 0.91 | 4.78 | 0.91 | 4.78 |
| AML | Null | 644 | 1.29 | 3.69 | 1.29 | 3.69 | 1.27 | 3.52 | 1.27 | 3.52 |
| AML | AD | 638 | 1.04 | 3.49 | 1.04 | 3.50 | 0.99 | 3.28 | 0.99 | 3.28 |
| AML | AP | 636 | 1.04 | 3.50 | 1.04 | 3.50 | 0.99 | 3.28 | 0.99 | 3.28 |
| AML | AC | 636 | 1.04 | 3.50 | 1.04 | 3.50 | 0.99 | 3.28 | 0.99 | 3.28 |
| AML | APC | 634 | 1.04 | 3.49 | 1.04 | 3.49 | 0.99 | 3.28 | 0.99 | 3.28 |
| HK | Null | 627 | 2.19 | 4.49 | 2.19 | 4.49 | 2.66 | 3.71 | 2.66 | 3.71 |
| HK | AD | 621 | 1.06 | 3.19 | 4.50 | 3.25 | 1.05 | 2.42 | 1.07 | 2.43 |
| HK | AP | 619 | 1.07 | 3.19 | 5.30 | 3.25 | 1.06 | 2.42 | 1.07 | 2.44 |
| HK | AC | 619 | 1.07 | 3.19 | 4.56 | 3.25 | 1.04 | 2.41 | 1.04 | 2.43 |
| HK | APC | 617 | 1.08 | 3.19 | 5.14 | 3.26 | 1.01 | 2.41 | 1.04 | 2.41 |
| NHK | Null | 627 | 1.28 | 4.34 | 1.28 | 4.34 | 0.78 | 3.07 | 0.78 | 3.07 |
| NHK | AD | 621 | 1.15 | 4.18 | 1.15 | 4.19 | 0.78 | 3.08 | 0.78 | 3.08 |
| NHK | AP | 619 | 1.11 | 4.16 | 1.11 | 4.16 | 0.78 | 3.08 | 0.78 | 3.08 |
| NHK | AC | 619 | 1.10 | 4.16 | 1.10 | 4.16 | 0.78 | 3.08 | 0.78 | 3.08 |
| NHK | APC | 617 | 1.06 | 4.14 | 1.06 | 4.14 | 0.78 | 3.09 | 0.78 | 3.09 |
| CNS | Null | 644 | 1.30 | 5.48 | 1.30 | 5.48 | 1.36 | 5.32 | 1.36 | 5.32 |
| CNS | AD | 638 | 1.12 | 5.32 | 1.12 | 5.32 | 1.10 | 5.08 | 1.10 | 5.08 |
| CNS | AP | 636 | 1.11 | 5.31 | 1.11 | 5.31 | 1.08 | 5.07 | 1.08 | 5.07 |
| CNS | AC | 636 | 1.12 | 5.32 | 1.12 | 5.32 | 1.09 | 5.08 | 1.09 | 5.08 |
| CNS | APC | 634 | 1.11 | 5.31 | 1.11 | 5.31 | 1.08 | 5.07 | 1.08 | 5.07 |
| Neuroblastomas | Null | 644 | 5.17 | 6.43 | 5.17 | 6.43 | 4.56 | 5.69 | 4.56 | 5.69 |
| Neuroblastomas | AD | 638 | 1.22 | 3.08 | 1.21 | 3.09 | 1.08 | 2.87 | 1.08 | 2.88 |
| Neuroblastomas | AP | 636 | 1.23 | 3.08 | 1.22 | 3.09 | 1.07 | 2.87 | 1.08 | 2.88 |
| Neuroblastomas | AC | 636 | 1.22 | 3.09 | 1.21 | 3.09 | 1.08 | 2.88 | 1.09 | 2.88 |
| Neuroblastomas | APC | 634 | 1.18 | 3.05 | 1.19 | 3.06 | 1.06 | 2.86 | 1.07 | 2.86 |
| Retinoblastomas | Null | 644 | 5.12 | 4.48 | 5.12 | 4.48 | 4.87 | 4.31 | 4.87 | 4.31 |
| Retinoblastomas | AD | 638 | 1.03 | 1.74 | 1.02 | 1.74 | 0.98 | 1.60 | 8.70 | 1.64 |
| Retinoblastomas | AP | 636 | 1.03 | 1.74 | 1.03 | 1.75 | 0.98 | 1.60 | 8.62 | 1.64 |
| Retinoblastomas | AC | 636 | 1.03 | 1.74 | 1.03 | 1.74 | 0.96 | 1.60 | 9.52 | 1.64 |
| Retinoblastomas | APC | 634 | 1.03 | 1.74 | 1.05 | 1.74 | 1.38 | 1.59 | 3.26 | 1.63 |
| Renal | Null | 644 | 3.36 | 5.20 | 3.36 | 5.20 | 3.40 | 5.28 | 3.40 | 5.28 |
| Renal | AD | 638 | 1.00 | 2.93 | 1.00 | 2.93 | 1.05 | 2.99 | 1.05 | 2.99 |
| Renal | AP | 636 | 1.00 | 2.93 | 1.00 | 2.93 | 1.06 | 3.00 | 1.06 | 3.00 |
| Renal | AC | 636 | 1.00 | 2.93 | 1.00 | 2.93 | 1.06 | 3.00 | 1.06 | 3.00 |
| Renal | APC | 634 | 1.00 | 2.94 | 1.00 | 2.94 | 1.06 | 3.00 | 1.06 | 3.00 |
| Hepatic | Null | 644 | 1.98 | 1.84 | 1.98 | 1.84 | 1.86 | 1.50 | 1.86 | 1.50 |
| Hepatic | AD | 638 | 1.10 | 1.23 | 1.13 | 1.23 | 1.00 | 0.91 | 1.04 | 0.92 |
| Hepatic | AP | 636 | 1.10 | 1.23 | 1.13 | 1.24 | 0.99 | 0.92 | 1.02 | 0.92 |
| Hepatic | AC | 636 | 1.11 | 1.23 | 1.12 | 1.24 | 0.96 | 0.92 | 0.95 | 0.92 |
| Hepatic | APC | 634 | 1.09 | 1.24 | 1.07 | 1.24 | 0.96 | 0.92 | 0.94 | 0.92 |
| Bone | Null | 644 | 2.25 | 4.25 | 2.25 | 4.25 | 2.17 | 4.05 | 2.17 | 4.05 |
| Bone | AD | 638 | 1.02 | 2.94 | 1.02 | 2.94 | 1.06 | 2.89 | 1.06 | 2.89 |
| Bone | AP | 636 | 1.02 | 2.95 | 1.02 | 2.95 | 1.05 | 2.88 | 1.05 | 2.88 |
| Bone | AC | 636 | 1.02 | 2.95 | 1.02 | 2.95 | 1.06 | 2.89 | 1.06 | 2.89 |
| Bone | APC | 634 | 1.02 | 2.95 | 1.02 | 2.95 | 1.05 | 2.88 | 1.05 | 2.88 |
| Soft tissue | Null | 644 | 1.25 | 4.11 | 1.25 | 4.11 | 1.20 | 3.68 | 1.20 | 3.68 |
| Soft tissue | AD | 638 | 1.05 | 3.92 | 1.05 | 3.91 | 1.00 | 3.50 | 1.00 | 3.50 |
| Soft tissue | AP | 636 | 1.05 | 3.92 | 1.05 | 3.92 | 0.98 | 3.50 | 0.98 | 3.50 |
| Soft tissue | AC | 636 | 1.04 | 3.91 | 1.04 | 3.92 | 0.98 | 3.50 | 0.98 | 3.50 |
| Soft tissue | APC | 634 | 1.04 | 3.92 | 1.04 | 3.92 | 0.98 | 3.50 | 0.98 | 3.50 |
| Germ cells | Null | 644 | 2.32 | 3.44 | 2.32 | 3.44 | 1.72 | 3.32 | 1.72 | 3.32 |
| Germ cells | AD | 638 | 1.16 | 2.57 | 1.15 | 2.57 | 1.12 | 2.82 | 1.12 | 2.82 |
| Germ cells | AP | 636 | 1.16 | 2.57 | 1.16 | 2.57 | 1.12 | 2.83 | 1.12 | 2.83 |
| Germ cells | AC | 636 | 1.12 | 2.54 | 1.12 | 2.54 | 1.12 | 2.83 | 1.11 | 2.83 |
| Germ cells | APC | 634 | 1.10 | 2.52 | 1.09 | 2.52 | 1.12 | 2.83 | 1.12 | 2.83 |
| Epithelial | Null | 644 | 1.85 | 3.45 | 1.85 | 3.45 | 2.23 | 3.98 | 2.23 | 3.98 |
| Epithelial | AD | 638 | 0.98 | 2.69 | 0.98 | 2.69 | 1.10 | 3.01 | 1.11 | 3.01 |
| Epithelial | AP | 636 | 0.96 | 2.67 | 0.96 | 2.67 | 1.07 | 2.99 | 1.07 | 2.99 |
| Epithelial | AC | 636 | 0.95 | 2.66 | 0.95 | 2.66 | 1.09 | 3.01 | 1.09 | 3.01 |
| Epithelial | APC | 634 | 0.95 | 2.66 | 0.96 | 2.66 | 1.04 | 2.96 | 1.04 | 2.97 |

1. **Model fit indices: adolescents and young adults (age 15-24 years)**

|  | | | **Males** | | | | **Females** | | | |
| --- | --- | --- | --- | --- | --- | --- | --- | --- | --- | --- |
|  |  |  | **LOG** | | **POWER** | | **LOG** | | **POWER** | |
|  | **Model** | **df** | **Chi^2^** | **AIC** | **Chi^2^** | **AIC** | **Chi^2^** | **AIC** | **Chi^2^** | **AIC** |
| Leukemia | Null | 429 | 1.072 | 5.028 | 1.072 | 5.028 | 1.005 | 4.551 | 1.005 | 4.551 |
| Leukemia | AD | 423 | 0.967 | 4.933 | 0.967 | 4.933 | 0.953 | 4.512 | 0.953 | 4.512 |
| Leukemia | AP | 421 | 0.971 | 4.941 | 0.971 | 4.940 | 0.956 | 4.520 | 0.956 | 4.520 |
| Leukemia | AC | 421 | 0.972 | 4.942 | 0.972 | 4.942 | 0.957 | 4.522 | 0.957 | 4.521 |
| Leukemia | APC | 419 | 0.973 | 4.948 | 0.973 | 4.948 | 0.957 | 4.527 | 0.957 | 4.527 |
| HL | Null | 429 | 1.797 | 6.190 | 1.797 | 6.190 | 1.477 | 5.750 | 1.477 | 5.750 |
| HL | AD | 423 | 1.063 | 5.434 | 1.064 | 5.435 | 1.003 | 5.239 | 1.001 | 5.238 |
| HL | AP | 421 | 1.049 | 5.424 | 1.051 | 5.425 | 0.964 | 5.204 | 0.963 | 5.202 |
| HL | AC | 421 | 1.030 | 5.410 | 1.031 | 5.411 | 0.951 | 5.193 | 0.950 | 5.191 |
| HL | APC | 419 | 1.024 | 5.408 | 1.024 | 5.407 | 0.953 | 5.200 | 0.952 | 5.198 |
| NHL | Null | 429 | 1.341 | 4.914 | 1.341 | 4.914 | 1.206 | 4.162 | 1.206 | 4.162 |
| NHL | AD | 423 | 1.174 | 4.782 | 1.173 | 4.782 | 0.945 | 3.924 | 0.947 | 3.926 |
| NHL | AP | 421 | 1.158 | 4.777 | 1.158 | 4.778 | 0.944 | 3.929 | 0.946 | 3.931 |
| NHL | AC | 421 | 1.159 | 4.776 | 1.159 | 4.777 | 0.947 | 3.931 | 0.950 | 3.933 |
| NHL | APC | 419 | 1.162 | 4.785 | 1.162 | 4.785 | 0.937 | 3.931 | 0.937 | 3.930 |
| CNS | Null | 429 | 1.391 | 5.447 | 1.391 | 5.447 | 1.346 | 5.129 | 1.346 | 5.129 |
| CNS | AD | 423 | 1.191 | 5.245 | 1.191 | 5.245 | 1.184 | 5.004 | 1.185 | 5.005 |
| CNS | AP | 421 | 1.187 | 5.248 | 1.187 | 5.248 | 1.190 | 5.012 | 1.191 | 5.013 |
| CNS | AC | 421 | 1.187 | 5.247 | 1.187 | 5.246 | 1.189 | 5.012 | 1.190 | 5.013 |
| CNS | APC | 419 | 1.190 | 5.254 | 1.190 | 5.254 | 1.194 | 5.019 | 1.196 | 5.020 |
| Bone | Null | 429 | 1.351 | 4.666 | 1.351 | 4.666 | 1.367 | 4.141 | 1.367 | 4.141 |
| Bone | AD | 423 | 1.016 | 4.349 | 1.015 | 4.348 | 1.058 | 3.864 | 1.058 | 3.864 |
| Bone | AP | 421 | 1.014 | 4.351 | 1.013 | 4.351 | 1.052 | 3.867 | 1.051 | 3.865 |
| Bone | AC | 421 | 1.013 | 4.351 | 1.013 | 4.351 | 1.050 | 3.864 | 1.049 | 3.863 |
| Bone | APC | 419 | 1.018 | 4.360 | 1.017 | 4.359 | 1.053 | 3.866 | 1.052 | 3.866 |
| Soft tissue | Null | 429 | 1.062 | 4.181 | 1.062 | 4.181 | 1.223 | 4.160 | 1.223 | 4.160 |
| Soft tissue | AD | 423 | 1.034 | 4.174 | 1.034 | 4.174 | 1.162 | 4.109 | 1.162 | 4.109 |
| Soft tissue | AP | 421 | 1.037 | 4.180 | 1.037 | 4.181 | 1.150 | 4.107 | 1.149 | 4.106 |
| Soft tissue | AC | 421 | 1.036 | 4.178 | 1.036 | 4.178 | 1.148 | 4.103 | 1.148 | 4.103 |
| Soft tissue | APC | 419 | 1.037 | 4.183 | 1.037 | 4.183 | 1.143 | 4.110 | 1.144 | 4.110 |
| Germ cells | Null | 429 | 8.377 | 12.959 | 8.377 | 12.959 | 1.092 | 3.576 | 1.092 | 3.576 |
| Germ cells | AD | 423 | 1.045 | 5.474 | 1.041 | 5.466 | 1.078 | 3.566 | 1.078 | 3.566 |
| Germ cells | AP | 421 | 0.966 | 5.406 | 0.977 | 5.411 | 1.082 | 3.571 | 1.081 | 3.571 |
| Germ cells | AC | 421 | 0.986 | 5.427 | 0.975 | 5.414 | 1.077 | 3.565 | 1.077 | 3.565 |
| Germ cells | APC | 419 | 0.968 | 5.412 | 0.974 | 5.416 | 1.077 | 3.572 | 1.076 | 3.571 |
| Melanomas | Null | 429 | 3.807 | 7.133 | 3.807 | 7.133 | 6.579 | 10.180 | 6.579 | 10.180 |
| Melanomas | AD | 423 | 1.085 | 4.611 | 1.092 | 4.609 | 1.345 | 5.320 | 1.372 | 5.333 |
| Melanomas | AP | 421 | 1.033 | 4.554 | 1.054 | 4.567 | 1.201 | 5.178 | 1.259 | 5.219 |
| Melanomas | AC | 421 | 1.039 | 4.563 | 1.045 | 4.560 | 1.175 | 5.167 | 1.196 | 5.172 |
| Melanomas | APC | 419 | 1.037 | 4.563 | 1.038 | 4.564 | 1.181 | 5.172 | 1.182 | 5.173 |
| Carcinomas | Null | 429 | 2.341 | 6.117 | 2.341 | 6.117 | 10.163 | 13.536 | 10.163 | 13.536 |
| Carcinomas | AD | 423 | 1.165 | 4.932 | 1.164 | 4.931 | 1.286 | 5.821 | 1.320 | 5.852 |
| Carcinomas | AP | 421 | 1.062 | 4.837 | 1.057 | 4.831 | 1.131 | 5.682 | 1.152 | 5.701 |
| Carcinomas | AC | 421 | 1.078 | 4.846 | 1.082 | 4.849 | 1.189 | 5.732 | 1.243 | 5.782 |
| Carcinomas | APC | 419 | 1.067 | 4.844 | 1.062 | 4.839 | 1.127 | 5.685 | 1.125 | 5.683 |
