# Supplementary material for: Cancer incidence in English children, adolescents and young people: past trends and projections to 2030
Source: Br J Cancer. 2017 Nov 2;117(12):1865–73. doi: 10.1038/bjc.2017.341 (PMC5729467; doi:10.1038/bjc.2017.341)
Supplement: Supplementary Table 2 [file bjc2017341x2.docx]

**SUPPLEMENTARY TABLE 2**

Number of cancer estimated using the APC-model approach (i.e., modelled) vs. number obtained by multiplying the population projections for 2030 by the rate for the most recent available years (i.e., rate averaged across 2011-2013) vs. umber obtained by multiplying population estimates for 2013 by the projected rate in 2030

1. Children (age-0-14 years)

| **Sex** | **Cancer** | **Primary** | **Rate2013***  **pop2030** | **Rate2030***  **pop2013** |
| --- | --- | --- | --- | --- |
| Males | ALL | 221 | 212 | 206 |
|  | AML | 44 | 41 | 40 |
|  | HL | 49 | 46 | 42 |
|  | NHL | 77 | 72 | 69 |
|  | CNS | 220 | 205 | 200 |
|  | Neuroblastoma | 49 | 48 | 48 |
|  | Retinoblastoma | 22 | 22 | 22 |
|  | Renal | 42 | 41 | 41 |
|  | Hepatic | 12 | 10 | 12 |
|  | Osseous | 35 | 34 | 30 |
|  | Soft tissue | 67 | 61 | 61 |
|  | Germ cells | 26 | 24 | 25 |
|  | Epithelial | 25 | 25 | 22 |
|  | Other & unspecified | 5 | 6 | 5 |
| Females | ALL | 185 | 172 | 173 |
|  | AML | 39 | 36 | 36 |
|  | HL | 37 | 30 | 32 |
|  | NHL | 31 | 30 | 28 |
|  | CNS | 194 | 177 | 177 |
|  | Neuroblastoma | 47 | 44 | 46 |
|  | Retinoblastoma | 23 | 22 | 23 |
|  | Renal | 50 | 46 | 48 |
|  | Hepatic | 8 | 6 | 7 |
|  | Osseous | 31 | 31 | 27 |
|  | Soft tissue | 49 | 45 | 45 |
|  | Germ cells | 35 | 30 | 32 |
|  | Epithelial | 31 | 30 | 27 |
|  | Other & unspecified | 6 | 6 | 6 |

1. Adolescents and young adults (age 15-24 years)

| **Sex** | **Cancer** | **Primary** | **Rate2013***  **pop2030** | **Rate2030***  **pop2013** |
| --- | --- | --- | --- | --- |
| Males | Leukemia | 102 | 100 | 95 |
|  | HL | 170 | 150 | 159 |
|  | NHL | 76 | 77 | 71 |
|  | CNS | 133 | 123 | 124 |
|  | Osseous | 62 | 59 | 57 |
|  | Soft tissue | 50 | 47 | 46 |
|  | Germ cells | 257 | 258 | 244 |
|  | Carcinoma | 162 | 119 | 153 |
|  | Melanoma | 167 | 127 | 158 |
|  | Other & specified | 43 | 40 | 41 |
| Females | Leukemia | 65 | 63 | 1 |
|  | HL | 185 | 146 | 176 |
|  | NHL | 49 | 47 | 47 |
|  | CNS | 99 | 93 | 94 |
|  | Osseous | 36 | 35 | 34 |
|  | Soft tissue | 45 | 42 | 43 |
|  | Germ cells | 33 | 29 | 32 |
|  | Carcinoma | 426 | 301 | 415 |
|  | Melanoma | 276 | 206 | 268 |
|  | Other & specified | 43 | 39 | 41 |
