# Supplementary figures and images for: Cancer incidence in English children, adolescents and young people: past trends and projections to 2030
Source: Br J Cancer. 2017 Nov 2;117(12):1865–73. doi: 10.1038/bjc.2017.341 (PMC5729467; doi:10.1038/bjc.2017.341)

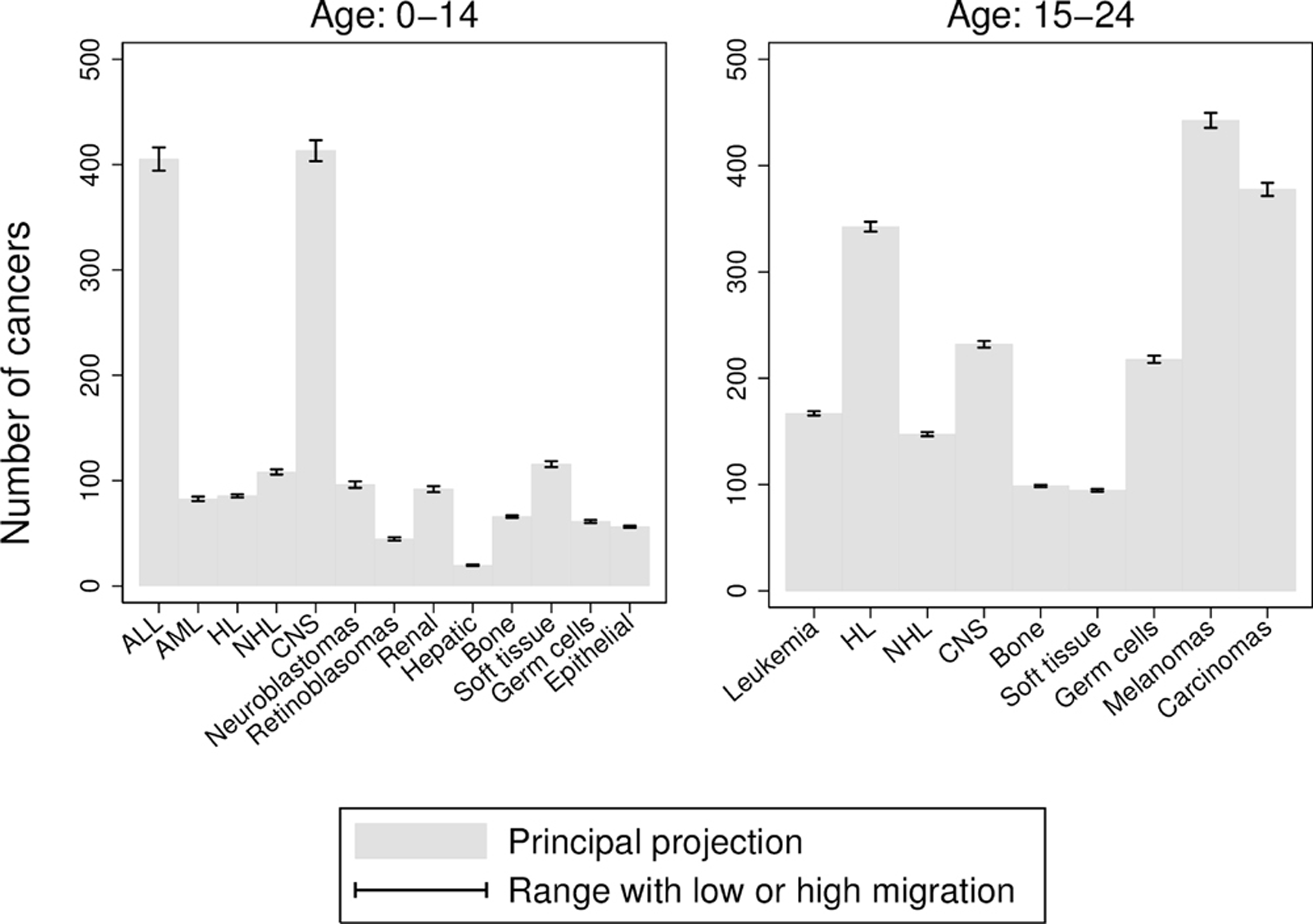

Supplement: Supplementary Figure 1 [file bjc2017341x3.tif]

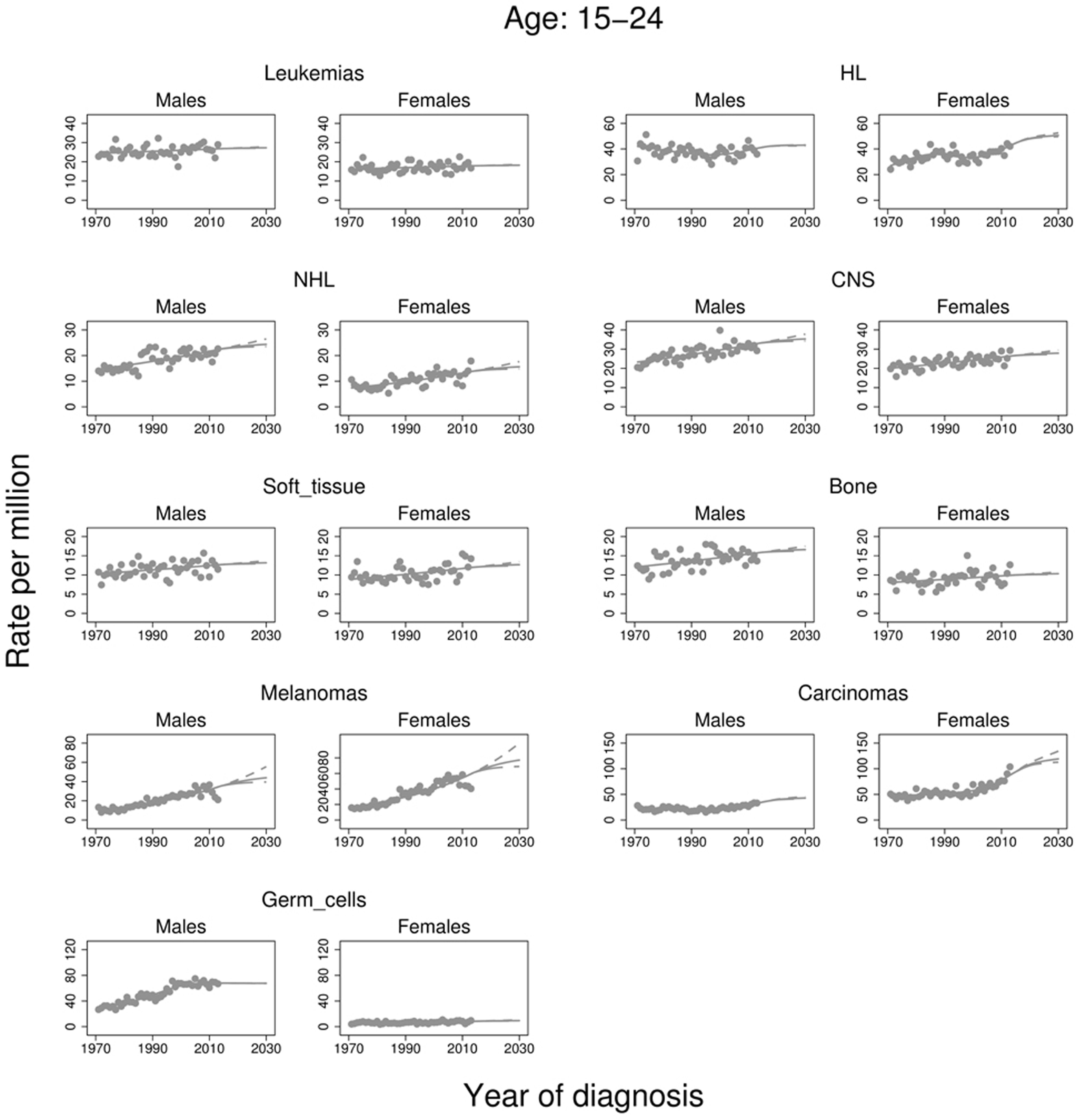

Supplement: Supplementary Figure 3 [file bjc2017341x5.tif]
